# Quantification of cytosine modifications in the aged mouse brain

**DOI:** 10.1002/npr2.12396

**Published:** 2023-12-06

**Authors:** Hiroko Sugawara, Akitoshi Date, Satoshi Fuke, Yutaka Nakachi, Tadafumi Kato, Minoru Narita, Miki Bundo, Kazuya Iwamoto

**Affiliations:** ^1^ Department of Psychiatry, Faculty of Medicine Fukuoka University Fukuoka Japan; ^2^ Department of Psychiatry Kansai Rosai Hospital Amagasaki Japan; ^3^ Department of Psychiatry, Graduate School of Medicine Osaka University Osaka Japan; ^4^ Department of Pharmacology Hoshi University School of Pharmacy and Pharmaceutical Sciences Tokyo Japan; ^5^ Lab for Molecular Dynamics of Mental Disorders RIKEN Center for Brain Science Wako Japan; ^6^ Research Unit/Neuroscience Sohyaku. Innovative Research Division, Mitsubishi Tanabe Pharma Corporation Yokohama Japan; ^7^ Department of Molecular Brain Science, Graduate School of Medical Sciences Kumamoto University Kumamoto Japan; ^8^ Department of Psychiatry and Behavior Science, Graduate School of Medicine Juntendo University Tokyo Japan

**Keywords:** 5‐formylcytosine: 5‐fC, 5‐hydroxymethycytosine: 5‐hmC, 5‐methylcytosine: 5‐mC, aging, basal ganglia

## Abstract

Quantifying cytosine modifications in various brain regions provides important insights into the gene expression regulation and pathophysiology of neuropsychiatric disorders. In this study, we quantified 5‐methylcytosine (5‐mC), 5‐hydroxymethylation (5‐hmC), and 5‐formylcytosine (5‐fC) levels in five brain regions (the frontal lobe, cerebral cortical region without frontal lobe, hippocampus, basal ganglia, and the cerebellum) and the heart at three developmental periods (12, 48, and 101 weeks). We observed significant regional variations in cytosine modification. Notably, regional variations were generally maintained throughout development, suggesting that epigenetic regulation is unique to each brain region and remains relatively stable with age. The 5‐mC and 5‐hmC levels were positively correlated, although the extent of the correlations seemed to differ in different brain regions. On the contrary, 5‐fC levels did not correlate with 5‐mC or 5‐hmC levels. Additionally, we observed an age‐dependent decrease in 5‐fC levels in the basal ganglia, suggesting a unique epigenetic regulation mechanism. Further high‐resolution studies using animal models of neuropsychiatric disorders as well as postmortem brain evaluation are warranted.

## Graphical abstract

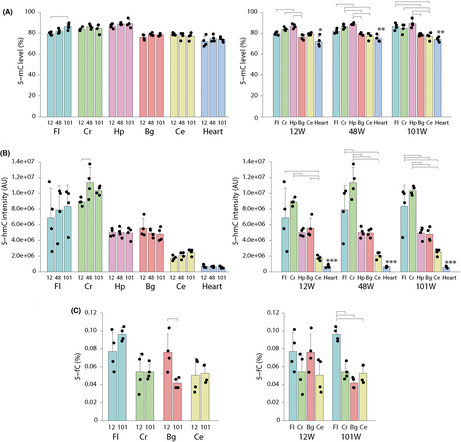

## 1 INTRODUCTION

Epigenetics is the study of the genetic regulation of gene expression in which DNA sequences are not altered. DNA methylation is a major form of DNA modification and contributes to long‐lasting alterations in gene expression.^1^ This process can be affected by environmental factors and has been considered to reflect gene–environment interactions.^2^ Numerous studies have suggested that DNA methylation plays an important role in brain development and the pathophysiology of neuropsychiatric disorders.^3^, ^4^

Although DNA methylation at the 5′ position of cytosine (5‐methylcytosine: 5‐mC) has been studied for decades, other modifications such as 5‐hydroxymethylation (5‐hmC) have recently attracted significant attention.^5^, ^6^ In addition, researchers have identified multiple oxidized 5‐mC variants, which are the products of sequential oxidation of 5‐mC to 5‐hmC, 5‐hmC to 5‐formylcytosine (5‐fC), and 5‐fC to 5‐carboxylcytosine by Ten‐eleven translocation (TET) enzymes.^7^, ^8^ The functional significance of cytosine modifications has not yet been fully elucidated; however, it has been suggested that they serve a unique regulatory function in active DNA demethylation in the brain.^9^, ^10^

In mammals, global 5‐hmC content varies between tissues in the highest levels of the brain.^11^, ^12^ In mice, 5‐mC and 5‐hmC content in brain tissue is increased during early postnatal development and stabilizes after approximately 10 weeks,^13^, ^14^, ^15^, ^16^ although timing differs between 5‐mC and 5‐hmC and between the cerebellum and cortex.^17^ In contrast, 5‐fC levels drastically decrease after birth until postnatal Day 14, after which its level seems to stabilize in the mouse cerebral cortex.^18^

Quantifying cytosine modifications in various brain regions will provide important insights into the gene expression regulation and pathophysiology of neuropsychiatric disorders such as bipolar disorder, schizophrenia, and Alzheimer's disorders, in which variations in global cytosine modifications have been frequently reported.^19^, ^20^, ^21^, ^22^ Few studies have conducted systematic analyses of cytosine modifications of multiple brain regions at different developmental stages. In this study, we quantified 5‐mC, 5‐hmC, and 5‐fC levels in various brain regions of adult mice and focused on aging‐related changes to these levels throughout development.

## 2 METHODS

### 2.1 Animals

Two pairs of male and female C57BL/6 mice, aged 12, 48, and 101 weeks, were used for the experiment. Genomic DNA was isolated from five brain regions (frontal lobes, Fl; cerebral cortical regions without the frontal lobe, Cr; hippocampus, Hp; basal ganglia, Bg; and cerebellum, Ce), and the heart. All experimental procedures involving animals were approved by the Animal Experiment Committees of RIKEN (Wako, Saitama, Japan) and were conducted in accordance with the Guidelines for Proper Conduct of Animal Experiments by the Science Council of Japan.

### 2.2 Measurement of global 5‐mC level

To quantify the global 5‐mC level, the luminometric methylation assay (LUMA)^23^ was used with slight modifications.^24^ Genomic DNA was digested with EcoRI (New England Biolabs, Beverly, MA, USA) and either HpaII (New England Biolabs) or MspI (New England Biolabs). The enzymes HpaII and MspI are isoschizomers that recognize the CCGG sequence and cleave CG. MspI is a CpG methylation‐insensitive restriction endonuclease whereas HpaII is sensitive to CpG methylation. After digestion, the 5′‐CG overhang was quantified by a luminometric polymerase extension assay using a PSQ 96MA instrument (QIAGEN, Hilden, Germany) according to the manufacturer's instructions. The relative amount of DNA methylation is expressed as the HpaII/MspI ratio, which indicates the amount of CpG methylation (5‐mC and 5‐hmC, not including 5‐fC) in the context of CCGG. The amount of DNA used for each restriction cleavage reaction was 500 ng, and each assay was replicated once. In each assay, methylation level was determined based on the standard curves using the samples containing various amounts of 100% methylated and unmethylated genomic DNA using 5‐Methylcytosine & 5‐Hydroxymethylcytosine DNA Standard Set (Zymo Research, Irvine, CA, USA).

### 2.3 Measurement of 5‐hmC level

A dot blot assay was performed to calculate 5‐hmC signal intensity. Genomic DNA was measured using a Qubit dsDNA HS assay kit (Invitrogen, Carlsbad, CA, USA). Five nanograms of DNA was denatured in Tris‐EDTA at 95°C for 10 min and spotted on a Zeta‐Probe GT (Genomic Tested) Blotting Membranes (Bio‐Rad Laboratories, Hercules, CA, USA) using Minifold I Spot‐Blot System (Whatman plc, Maidstone, UK). The membrane was blocked in blocking buffer (5% skim milk, TBS‐0.05% Tween‐20) at room temperature for 60 min and incubated with anti‐5‐hydroxymethylcytosine antibody (1:10 000) (Active Motif, Carlsbad, CA) at 4°C overnight. Horseradish peroxidase enzyme‐conjugated anti‐rabbit secondary antibody (1:2000) was incubated with the membrane at room temperature for 60 min, followed by washing with TBS‐T. The bound antibodies were visualized using enhanced chemiluminescence (GE Healthcare, Chicago, IL, USA). The membrane was imaged using ImageQuant LAS4000 (GE Healthcare) and quantification was performed using the ImageQuant TL system (GE Healthcare). In each assay, linearity of signals was ensured by including the standard 5‐hmC DNA samples (0.001, 0.01, 0.1, and 1 ng) using 5‐Methylcytosine & 5‐Hydroxymethylcytosine DNA Standard Set.

### 2.4 Measurement of global 5‐fC level

The assay was performed using a MethylFlash 5‐Formylcytosine DNA Quantification Kit (Epigentek, Farmingdale, NY, USA). In this assay, Fl, Cr, Bg, and Ce from 12‐ and 101‐week aged mice were analyzed due to the lack of available materials. The amount of genomic DNA was measured using a Qubit dsDNA BR assay kit (Invitrogen). One hundred nanograms of DNA as well as the negative and positive controls were incubated in the binding solution in each well at 37°C for 90 min. Subsequently, they were incubated with the 5‐fC capture antibody at room temperature for 60 min, and with the detection antibody at room temperature for 30 min. After color development using enhancer and developer solutions, absorbance was measured using a 96 plate‐reader at 450 nm. In each assay, linearity of signals was ensured by including the standard DNA samples provided by the manufacturer.

### 2.5 Statistics

Data were analyzed using correlation analysis and analysis of variance (ANOVA) followed by Tukey's multiple comparison test as a post hoc analysis. Differences were considered statistically significant at *p* < 0.05. Bell Curve for Excel (Social Survey Research Information Co., Ltd., Tokyo, Japan) was used for the statistical analyses.

## 3 RESULTS

We quantified the levels of cytosine modifications (5‐mC, 5‐hmC, and 5‐fC) in five brain regions (Fl, Cr, Hp, Bg, and Ce) and the heart at three developmental periods (12, 48, and 101 weeks). Owing to the availability of materials, we quantified 5‐fC levels in only four brain regions (Fl, Cr, Bg, and Ce) at two periods (12 and 101 weeks) (Table S1).

### 3.1 5‐mC level

**FIGURE 1:**
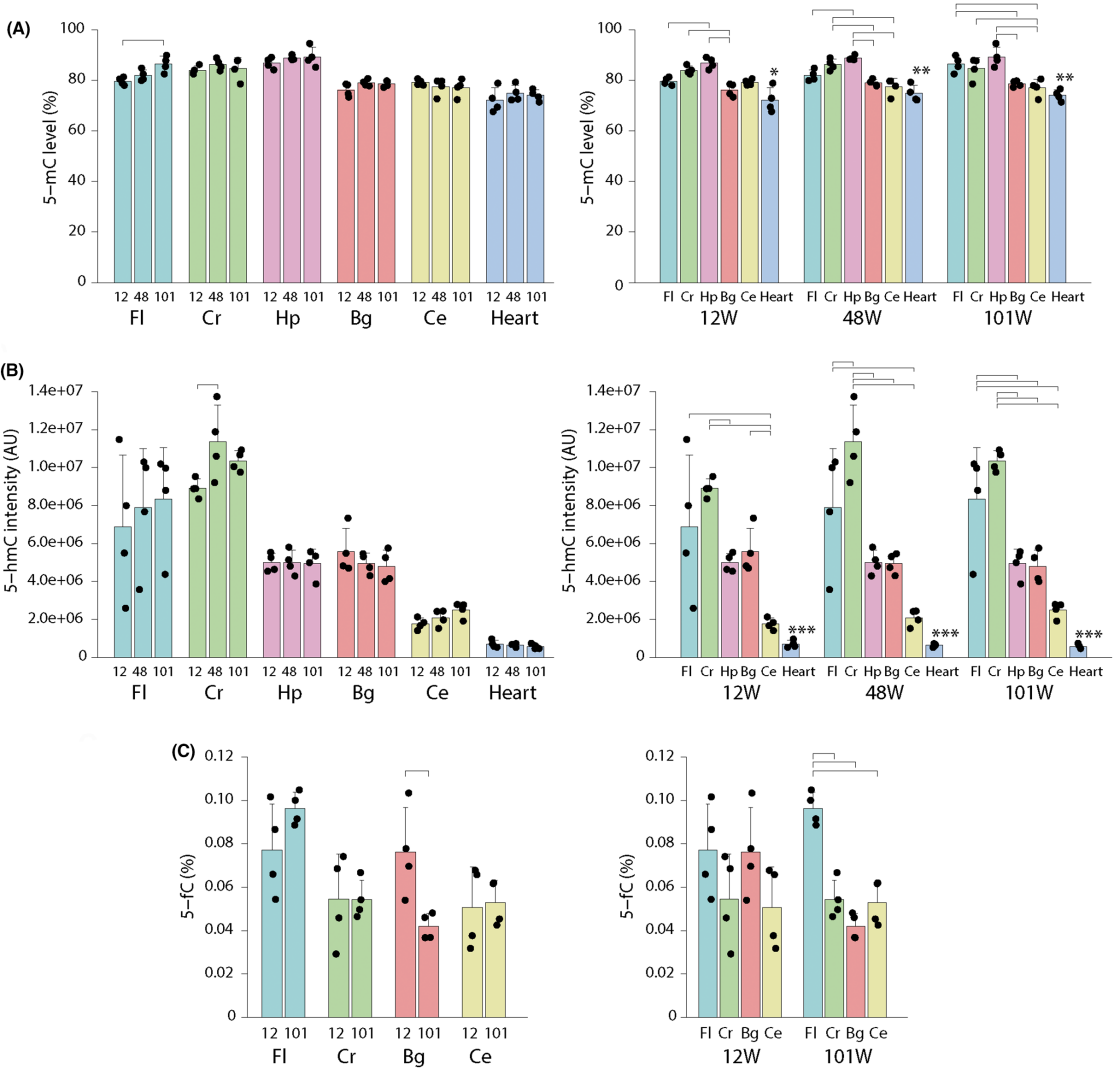
Cytosine modification levels of adult mouse brain. (A) 5‐mC, (B) 5‐hmC, and (C) 5‐fC levels. Left panels show comparisons of aging effect in each brain region. Right panels show comparisons across brain subregions and heart in each aging stage. In each group, four animals were used (two males and two females). Note that 5‐mC level includes both 5‐mC and 5‐hmC. Mean ± SD is shown. ANOVA followed by Tukey test (*p* < 0.05) is indicated. *, **, and *** indicate that the heart showed significance in all brain subregions except for Bg, both Bg and Ce, and Ce, respectively. Bg, basal ganglia; Ce, cerebellum; Cr, cerebral cortical regions without frontal lobe; Fl, frontal lobes; Hp, hippocampus.

The value obtained by LUMA reflected the total 5‐mC and 5‐hmC levels for 5′‐CCGG‐3′. In this study, this value was used as a proxy for the global 5‐mC level. Among the examined brain regions and the heart, only Fl showed a significant difference related to aging (ANOVA, *p* < 0.05). The post hoc test revealed a significant increase at 101 weeks compared with that at 12 weeks (Tukey, *p* < 0.05) (Figure 1A). We observed significant differences between brain regions and the heart and within brain regions. Among the brain regions, Hp showed the highest levels of methylation while Bg and Ce showed the lowest levels. The relationship between regional differences in 5‐mC was generally maintained throughout the course of aging.

### 3.2 5‐hmC level

There was a significant difference in Cr between Weeks 12 and 48 (ANOVA followed by Tukey's test, *p* < 0.05) (Figure 1B). The 5‐hmC signal intensities in all brain regions, except for Ce, were significantly higher than those in the heart throughout all stages. Among the brain regions, Ce showed the lowest level of 5‐hmC, and the cortical regions of Fc and Cr were higher than those in other brain regions. Similar to 5‐mC levels, the relationship between regional variations in 5‐hmC was maintained throughout all stages. However, the pattern was different between 5‐mC and 5‐hmC in that Hp and Bg showed the lowest levels and Ce showed the lowest levels compared to cortical regions in 5‐hmC.

### 3.3 5‐fC level

There was a significant difference in Bg between Weeks 12 and 101 (Figure 1C). At 101 weeks, the 5‐fC level in Fl was significantly higher than that in other brain regions.

### 3.4 Correlation analysis of cytosine modifications

**FIGURE 2:**
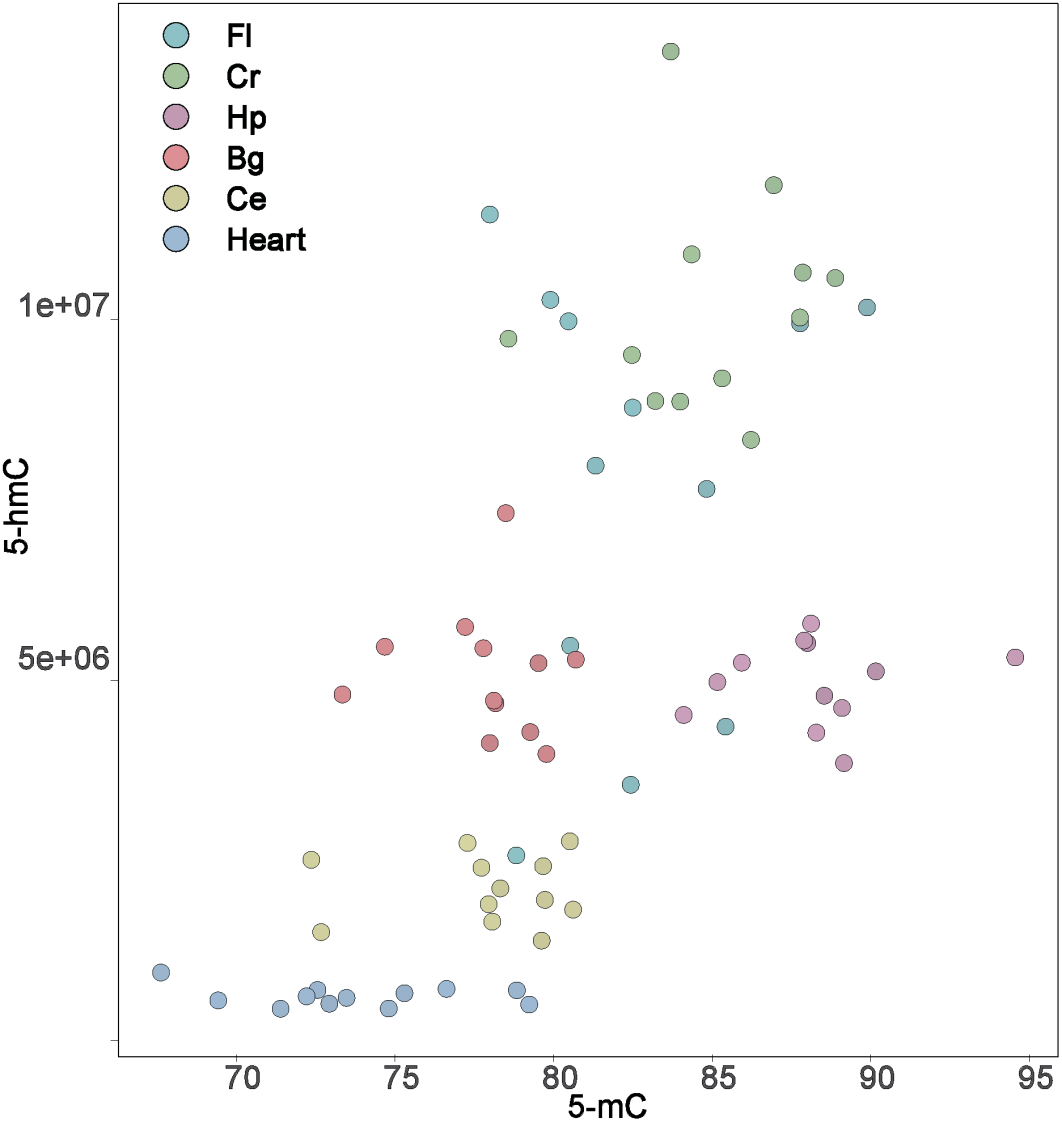
Correlation plot of 5‐mC and 5‐hmC levels. Note that 5‐mC level includes both 5‐mC and 5‐hmC.

When all data points were plotted, a significant correlation between 5‐mC and 5‐hmC levels was identified (Pearson's *R* = 0.57, *p* < 0.001; Figure 2). However, no significant correlations were detected between 5‐mC and 5‐fC or between 5‐hmC and 5‐fC (*p* > 0.05, data not shown). The exclusion of heart samples did not affect these correlations.

## 4 DISCUSSION

In this study, we quantified 5‐mC, 5‐hmC, and 5‐fC levels in multiple brain regions during different aging stages. In mice, drastic age‐related changes in cytosine modifications occurred at an early developmental stage.^13^, ^14^, ^15^, ^16^, ^17^, ^18^ Because our study focused on later developmental stages, fewer changes in modifications were expected in our assays compared to those reported in previous studies.

We observed significant regional variations in cytosine modification throughout the brain. Age‐dependent changes in global 5‐mC levels detected only in Fl may reflect both the prolonged development of this region and vulnerability to neurodegenerative pathology.^25^ Notably, regional variations were generally maintained throughout development, suggesting that epigenetic regulation is unique to each brain region and remains relatively stable with age. The concentration of neuronal cells, in which 5‐hmC is present prominently,^11^ could account for the differences in 5‐mC and 5‐hmC levels among the brain regions. The 5‐mC and 5‐hmC levels were positively correlated, although the extent of correlation seemed to differ by brain region and in the heart, suggesting that these modifications are closely linked. In contrast, 5‐fC levels did not correlate with 5‐mC or 5‐hmC levels. We identified aging‐related changes in 5‐fC levels: at 101 weeks, 5‐fC levels decreased in Bg. Bg consists of several subregions, such as the striatum, nucleus accumbens (NAc), and substantial nigra. Several studies have suggested distinct epigenetic regulation in these brain regions, such as altered TET expression in the NAc in response to rewards.^26^, ^27^ These results imply Bg‐specific epigenetic regulation of 5‐hmC to 5‐fC conversion, and gene expression analysis including TET would provide new insights.

This study was constrained by limitations. First, the LUMA measured both 5‐mC and 5‐hmC levels, thus complicating the interpretation of the results. Second, we were unable to compare the levels of the three cytosine modifications in each brain region because we employed independent assays with different principles. Third, because we used large amounts of frozen brain tissue, the resolution of the analysis was low. Therefore, we were unable to assess the cytosine modification status in Cr, Hp, and Bg subregions, and cell‐type differences were not considered. The latter is important because 5‐hmC accumulates in neurons,^17^ whereas 5‐fC is more enriched in non‐neuronal cells.^18^ Therefore, the identified age‐related changes in 5‐fC levels may be associated with altered composition of brain cell types. Fourth, with regard to sex, we compared the average 5‐mC, 5‐hmC, and 5‐fC levels between males and females. We did not observe large differences in 5‐mC or 5‐fC levels. In Fl, we observed higher 5‐hmC levels in females than in males. However, considering the limited number of samples with high interindividual variations, especially in 5‐hmC, more studies using enough numbers of animals will be required. Finally, although our study focused on later developmental stage of the brain, it is also important to investigate at an early developmental stage which is a critical period for psychiatric disorders such as schizophrenia and autism. Further high‐resolution studies using animal models of neuropsychiatric disorders or examinations of postmortem brains of patients are warranted.

## AUTHOR CONTRIBUTIONS

A.D., S.F., and M.B. performed experiments. H.S. and K.I. performed data analysis and wrote the manuscript. Y.N conducted data management. T.K., M.N., M.B., and K.I. designed this study. All authors have read and approved the final manuscript.

## FUNDING INFORMATION

This research was partly supported by JSPS KAKENHI under the grant numbers 18H05428, 18H02753, 18H05430, 18K15486, and 21K07548. This research was also partly supported by AMED under grant number JP19dm0207074.

## CONFLICT OF INTEREST STATEMENT

The authors declare that they have no competing interests. Author H.S. is an Editorial Board member of Neuropsychopharmacology Reports and a coauthor of this article. To minimize bias, they were excluded from all editorial decision‐making related to the acceptance of this article for publication. Author S.F is affiliated to Mitsubishi Tanabe Pharma Corporation. The company played no role in study design, data collection and analysis, decision to publish, and preparation of the manuscript.

## ETHICS STATEMENT

Approval of the research protocol by an Institutional Reviewer Board: N/A.

Informed Consent: N/A.

Registry and Registration No. of the study/trial: N/A.

Animal studies: All experimental procedures involving animals were approved by the Animal Experiment Committees of RIKEN (Wako, Saitama, Japan).

## Supporting information


Table S1

## Data Availability

The data that supports the findings of this study are available in the Table S1 of this article.
