# Supplementary material for: Quantification of cytosine modifications in the aged mouse brain
Source: Neuropsychopharmacol Rep. 2023 Dec 6;44(1):250–5. doi: 10.1002/npr2.12396 (PMC10932792; doi:10.1002/npr2.12396)
Supplement: Supplementary file 1 — Table S1 [file NPR2-44-250-s001.zip › npr212396-sup-0002-Table S1 caption.docx]

Table S1: The levels of cytosine modifications in five brain regions.
